# Multimodal MRI and ^31^P-MRS Investigations of the *ACTA1*(Asp286Gly) Mouse Model of Nemaline Myopathy Provide Evidence of Impaired *In Vivo* Muscle Function, Altered Muscle Structure and Disturbed Energy Metabolism

**DOI:** 10.1371/journal.pone.0072294

**Published:** 2013-08-20

**Authors:** Charlotte Gineste, Guillaume Duhamel, Yann Le Fur, Christophe Vilmen, Patrick J. Cozzone, Kristen J. Nowak, David Bendahan, Julien Gondin

**Affiliations:** 1 Aix-Marseille Université, Centre National de la Recherche Scientifique (CNRS), Centre de Résonance Magnétique Biologique et Médicale (CRMBM) Unité Mixte de Recherche (UMR) 7339, Marseille, France; 2 University of Western Australia, Centre for Medical Research, Western Australian Institute for Medical Research (WAIMR), Nedlands, Australia; University of Valencia, Spain

## Abstract

Nemaline myopathy (NM), the most common non-dystrophic congenital disease of skeletal muscle, can be caused by mutations in the skeletal muscle α-actin gene (*ACTA1*) (~25% of all NM cases and up to 50% of severe forms of NM). Muscle function of the recently generated transgenic mouse model carrying the human Asp286Gly mutation in the *ACTA1* gene (Tg(*ACTA1*)^Asp286Gly^) has been mainly investigated *in vitro*. Therefore, we aimed at providing a comprehensive picture of the *in vivo* hindlimb muscle function of Tg(*ACTA1*)^Asp286Gly^ mice by combining strictly noninvasive investigations. Skeletal muscle anatomy (hindlimb muscles, intramuscular fat volumes) and microstructure were studied using multimodal magnetic resonance imaging (Dixon, T_2_, Diffusion Tensor Imaging [DTI]). Energy metabolism was studied using 31-phosphorus Magnetic Resonance Spectroscopy (^31^P-MRS). Skeletal muscle contractile performance was investigated while applying a force-frequency protocol (1–150 Hz) and a fatigue protocol (6 min–1.7 Hz). Tg(*ACTA1*)^Asp286Gly^ mice showed a mild muscle weakness as illustrated by the reduction of both absolute (30%) and specific (15%) maximal force production. Dixon MRI did not show discernable fatty infiltration in Tg(*ACTA1*)^Asp286Gly^ mice indicating that this mouse model does not reproduce human MRI findings. Increased T_2_ values were observed in Tg(*ACTA1*)^Asp286Gly^ mice and might reflect the occurrence of muscle degeneration/regeneration process. Interestingly, T_2_ values were linearly related to muscle weakness. DTI experiments indicated lower λ_2_ and λ_3_ values in Tg(*ACTA1*)^Asp286Gly^ mice, which might be associated to muscle atrophy and/or the presence of histological anomalies. Finally ^31^P-MRS investigations illustrated an increased anaerobic energy cost of contraction in Tg(*ACTA1*)^Asp286Gly^ mice, which might be ascribed to contractile and non-contractile processes. Overall, we provide a unique set of information about the anatomic, metabolic and functional consequences of the Asp286Gly mutation that might be considered as relevant biomarkers for monitoring the severity and/or the progression of NM and for assessing the efficacy of potential therapeutic interventions.

## Introduction

Nemaline myopathy (NM) is the most common form of non-dystrophic congenital myopathies and is characterized by muscle dysfunction (almost always weakness) and the presence of rod shaped structures in the muscle fibers [1]. The clinical spectrum of NM has been divided into six different subtypes based, amongst several features, on the severity of the disease and the age of onset [2,3]. NM is a genetically heterogeneous muscular disorder caused by mutations in at least seven genes, most of them encoding for sarcomeric skeletal muscle thin filament proteins [4]. It is noteworthy that NM resulting from *ACTA1* mutations represents 15 to 25% of NM cases and up to 50% of the most severely affected patients [5,6].

Our understanding of the physiopathological mechanisms underlying muscle weakness in NM has been greatly improved due to the generation of both transgenic and knock-in mouse models of NM [7–9]. Over the last few years, four mouse models carrying mutations in the *ACTA1 or Acta1* genes and mimicking human NM have been generated [8,10–12]. The more recent model expresses the *ACTA1* (Asp286Gly) transgene containing a mutation previously identified in a patient with a severe form of NM [5]. This model has been generated either with [11] or without [10] an enhanced green fluorescent protein (EGFP)-tag and both reproduce the usual main clinical feature of NM, namely muscle weakness, as illustrated by the reduced maximal force production in isolated soleus and EDL muscles [10,11]. Investigations at the cross-bridges level also illustrated that this mutation prevents proper myosin cross-bridge binding and limits the fraction of actomyosin interactions in the strong binding state [13], thereby suggesting that the Asp286Gly mutation acts *in vitro* as a “poison-protein”. Although muscle function of nebulin-deficient mice was similarly altered at different levels (i.e., skinned muscle fibers, isolated muscles or muscle-tendon-bone unit) [14–16], other studies reported that *in vitro* alterations did not necessarily translate into similar changes *in vivo*, and vice versa [17,18]. On that basis, it becomes questionable as to whether and to what extent the Asp286Gly mutation affects the *in vivo* muscle function in Tg(*ACTA1*)^Asp286Gly^ mice.

The histological spectrum of the Tg(*ACTA1*)^Asp286Gly^ mice includes the presence of nemaline bodies, tubular aggregates and ringbinden fibers [10]. Additionally, a reduced fiber diameter indicating skeletal muscle atrophy has been reported in the version of this mouse model containing the EGFP-tag and has been associated with an ongoing chronic repair and regeneration process, as illustrated by the presence of fibers with central nuclei [11]. Considering this whole spectrum of functional and histological alterations, the multiple variables measured noninvasively using magnetic resonance imaging (MRI) could provide information of interest regarding the pathophysiological processes occurring in Tg(*ACTA1*)^Asp286Gly^ skeletal muscles. For instance, three-point Dixon technique, based on the chemical shift separation of water and fat signals, has been recently used for quantifying the extent of the fatty infiltration of dystrophic skeletal muscles [19–22]. Moreover, higher T_2_ values have been reported in *mdx* mice and have been related to various biological phenomena such as edema, necrosis, inflammation and fatty infiltration [23–25]. Finally, Diffusion Tensor Imaging (DTI) metrics (diffusivities and fractional anisotropy) have been linked to muscle degeneration/regeneration processes [26]. Overall, T_2_ mapping, DTI and 3-point Dixon measurements could provide relevant biomarkers of muscle degeneration/regeneration processes, muscle atrophy and muscular fatty infiltration in NM mouse muscles, respectively.

Abnormal glycogen accumulation as well as histological anomalies of the size and the shape of mitochondria have been observed in both *Acta1*(H40Y) mice [9] and in patients with typical or severe forms of NM [27,28]. An altered expression of several genes directly or indirectly involved in the glycolytic pathway has also been reported in NM patients [28]. On the basis of quantitative information related to high-energy phosphorylated compounds and pH in exercising muscle using 31-phosphorus Magnetic Resonance Spectroscopy (^31^P-MRS) [29–31], we recently demonstrated that the energy cost of contraction was higher for the *Acta1*(H40Y) mice as compared to controls [32]. Considering the multiple abnormalities reported so far in both *Acta1*(H40Y) mice and NM patients, we hypothesized that the Asp286Gly mutation could also disturb muscle energetics.

In the present study, we aimed at characterizing strictly noninvasively the functional, anatomical and metabolic consequences of the Asp286Gly mutation in a NM mouse model. Skeletal muscle contractile performance was investigated throughout a ramp frequency protocol in order to determine whether and to which extent this mutation affects the *in vivo* muscle function of Tg(*ACTA1*)^Asp286Gly^ mice. Additionally, several biomarkers of muscle atrophy, muscle degeneration/regeneration processes and muscular fatty infiltration were obtained by multimodal MRI while metabolic investigations were performed throughout a standardized fatiguing protocol using ^31^P-MRS.

## Materials and Methods

### Animals

Four-month old Tg(*ACTA1*)^Asp286Gly^ and wild-type (WT) mice were used for the experiments conducted in agreement with the French guidelines for animal care and in conformity with the European convention for the protection of vertebrate animals used for experimental purposes and institutional guidelines n° 86/609/CEE November 24, 1986. All animal experiments were approved by the Institutional Animal Care Committee of Aix-Marseille University (permit number: #15-14052012). Mice were housed in an environment-controlled facility (12-12 hour light-dark cycle, 22°C), received water and standard food *ad libitum*.

### Mechanical performance and ^31^P-MRS

Tg(*ACTA1*)^Asp286Gly^ (n = 9) and WT (n = 10) mice were tested twice over a 3-day period in order to assess mechanical performance and metabolic changes during a standardized stimulation protocol. During the first testing session, transcutaneous stimulation was first elicited with square-wave pulses (0.5 ms duration) on the gastrocnemius muscle. The individual maximal stimulation intensity was determined by progressively increasing the stimulus intensity until there was no further peak twitch force increase. This intensity was then maintained to elicit tetanic stimulations (duration = 0.75 sec; rest interval = 30 sec) at various incremental frequencies (from 1 to 150 Hz). During the second testing session, metabolic changes were investigated using ^31^P-MRS during a standardized stimulation protocol consisting of 6 min of repeated single twitch isometric contractions delivered at a frequency of 1.7 Hz [33].

#### Animal preparation

Mice were initially anesthetized in an induction chamber using 4% isoflurane in 33% O_2_ (0.5 L/min) and 66% N_2_O (1 L/min). The left hindlimb was shaved before an electrode cream was applied at the knee and heel regions to optimize electrical stimulation. Each anaesthetized mouse was placed supine in a home-built cradle which has been specially designed for the strictly noninvasive functional investigation of the left hindlimb muscles [33]. Throughout a typical experiment, anaesthesia was maintained by gas inhalation through a facemask continuously supplied with 1.75% isoflurane in 33% O_2_ (0.2 L/min) and 66% N_2_O (0.4 L/min). Exhaled and excess gases were removed through a canister filled with activated charcoal (Smiths Industries Medical System, Sheffield, UK) mounted on an electrical pump extractor (Equipement Vétérinaire Minerve, Esternay, France). Physiological temperature was adjusted with an electrical heating blanket. The foot was positioned on the pedal coupled to a force transducer. The pedal of the ergometer was adjusted to have a 90° flexion ankle joint. The hindlimb was centered inside a 20 mm-diameter ^1^H Helmholtz imaging coil and the belly of the gastrocnemius muscle was located above an elliptical (8 x 12 mm) ^31^P-MRS surface coil. Muscle contractions were achieved by transcutaneous electrical stimulation using two rod-shaped 1.5 mm-diameter surface electrodes integrated in the cradle and connected to an electrical stimulator (type 215/T; Hugo Sachs Elektronik-Harvard Apparatus GmbH, March-Hugstetten, Germany). One electrode was placed at the heel level and the other one was located just above the knee joint. The gastrocnemius muscle was chosen because it is easily accessible for ^31^P-MRS measurements and preferentially activated by our *in vivo* experimental set-up [33].

#### Force output measurements

Isometric force was measured with a home-built ergometer consisting of a 9 × 24 mm foot pedal coupled to a force transducer. The transducer was constructed by sticking a strain gauge (ref 1-LY11-6/120A; HBM GmbH, Darmstadt, Germany; 120-ohm internal resistance) on a Bakelite slat (0.4 mm thickness) in a Wheatstone bridge design (3 × 120 ohm). Electrically-evoked muscle contractions led to a deformation of the bakelite slat transmitted by the foot pedal resulting in a change in the strain gauge electrical resistance and a proportional voltage change. The resulting output signal was amplified with a home-built amplifier (Operational amplifier AD620; Analog Devices, Norwood, MA, USA; gain = 70 dB; bandwidth = 0-5 kHz) and converted to a digital signal (PCI-6220; National Instruments, Austin, TX, USA). It was continuously monitored and recorded on a personal computer using the WinATS software (Sysma, Aix-en-Provence, France).

#### ^31^P-MRS measurements

Metabolic investigations were performed in a 4.7-Tesla horizontal magnet (47/30 Biospec Avance, Bruker, Ettingen, Germany) equipped with a Bruker 120-mm (200 mT/m) gradient insert. Spectra (8-kHz sweep width; 2048 data points) from the gastrocnemius region were continuously acquired at rest and throughout the standardized stimulation protocol. A fully relaxed spectrum (12 accumulations, TR = 20 sec) was acquired at rest followed by a total of 256 free induction decays (FID) (TR = 1.875 sec). The first 64 FIDs were acquired at rest and summed together. The next 192 FIDs were acquired during the stimulation period and were summed by packets of 32, allowing a temporal resolution of ~ 60 sec. During the recovery period 512 FIDs were acquired. The first 224 FIDs were summed by packets of 32, allowing a temporal resolution of ~ 60 sec; the next 192 FIDs were summed by packets of 64, allowing a temporal resolution of ~ 120 sec; the last 96 FIDs were summed together, allowing a temporal resolution of ~ 180 sec.

### MR imaging

MRI experiments were initially performed on 6 Tg(*ACTA1*)^Asp286Gly^ and 8 WT mice for which mechanical performance and metabolic investigations were assessed (see above). Additional mice (n = 4 for Tg(*ACTA1*)^Asp286Gly^ and n = 3 for WT mice) were used to increase the sample size so that MRI experiments were performed on a total of 10 Tg(*ACTA1*)^Asp286Gly^ and 11 WT mice. Investigations were performed at 11.75 T on a vertical Bruker Avance 500 MHz/89mm wide-bore imager (Bruker, Ettlingen, Germany), equipped with high-performance actively shielded gradients (1 T/m maximum gradient strength, 9 kT/m/s maximum slew rate) and interfaced with Paravision 5.1. A transmit/receive volume RF coil (birdcage, diameter Ø = 3 cm, homogenous length L = 5 cm, Micro 2.5 Probe, Bruker, Ettlingen, Germany) was used for image acquisition.

#### Animal preparation

Mice were initially anesthetized in an induction chamber using 2% isoflurane and the anesthesia was maintained under spontaneous respiration (room airflow ~ 270 mL/min, regular breathing ~ 90 breaths/min) using 1.7% isoflurane (vaporizer Univentor 400 anesthesia unit, Zejtun, Malta). The respiration rate was controlled throughout the experiment using a compatible monitoring and gating system (SA Instruments, Stony Brook, NY). Physiological temperature was maintained by heating the gradient cooling system to 38°C. Mice were positioned head up in an animal bed and held using a teeth holder and taping their neck and pelvis to the cradle. The animal bed was then inserted into the RF coil with the hindlimb positioned at the center.

#### MRI sequences

Seven contiguous axial imaging slices (thickness = 1 mm) were selected across the left lower hindlimb based on a set of scout images. Three-point Dixon images were obtained with a three-dimensional gradient echo sequence using three different echo times for in-phase and opposed-phase imaging (TR = 189 ms; TE_1_ = 1.5; TE_2_ = 1.8 ms; TE_3_ = 2.1 ms; NEX = 4; flip angle = 25°; field of view = 2.0 x 2.0 cm^2^; matrix size = 128x 128). T_2_-weighted images (T_2_-w) were obtained using a 4-shot SE-EPI sequence with the following parameters: TR = 3500ms; 5 TEs (8, 12, 16, 20 and 24 ms); NEX = 15; field of view = 2.0 × 2.0 cm^2^; matrix = 128 × 128; acquisition time ^≈^ 20 min. DTI experiments were performed with a Stejskal-Tanner preparation and a segmented (4 shots) SE-EPI readout technique. The following acquisition parameters were used: diffusion gradient duration (δ) = 5 ms; time between diffusion gradients (Δ) = 10 ms; b-values = (0 and 450) sec/mm^2^ and 12 diffusion-encoding directions. Imaging parameters were: BW = 400 kHz; TR = 3500 ms; TE = 20 ms; NEX = 15; field of view = 2.0 x 2.0 cm^2^; matrix size = 128x 128. The total acquisition time was ~ 45 min. For both T_2_-w and DTI acquisitions, a fat suppression module was used.

### Data processing

#### Mechanical performance

For each stimulation train, isometric peak force was calculated and the corresponding data were fitted to the Hill equation providing f_50_ (stimulation frequency for which 50% of the maximal force was exerted). The maximum rate of force development (in mN/ms) and the half relaxation time, i.e. the time to obtain half of the decline in maximal tetanic force, were calculated from the tetanus obtained at 150 Hz. Regarding the standardized fatiguing stimulation protocol, the amplitude of each peak twitch was measured and was then averaged every 15 sec of stimulation. For all stimulation protocols, force was divided by the corresponding hindlimb muscles volume (see below) in order to obtain specific force (in mN/mm^3^).

#### MR data

^31^P-MRS: Data were processed using a proprietary software developed using IDL (Interactive Data Language, Research System, Inc., Boulder, CO, USA) [34]. Relative concentrations of phosphocreatine (PCr), inorganic phosphate (Pi) and ATP were obtained with a 60 sec time-resolution by a time-domain fitting routine using the AMARES-MRUI Fortran code and appropriate prior knowledge of the ATP multiplets. PCr to ATP ratios were calculated from the peak areas of the spectrum acquired at rest. Intracellular pH (pHi) was calculated from the chemical shift of the Pi signal relative to PCr [35]. PCr recovery kinetics after the stimulation protocol was fitted using a monoexponential fit and the PCr recovery time (τPCr) value was calculated.

**Figure 1:**
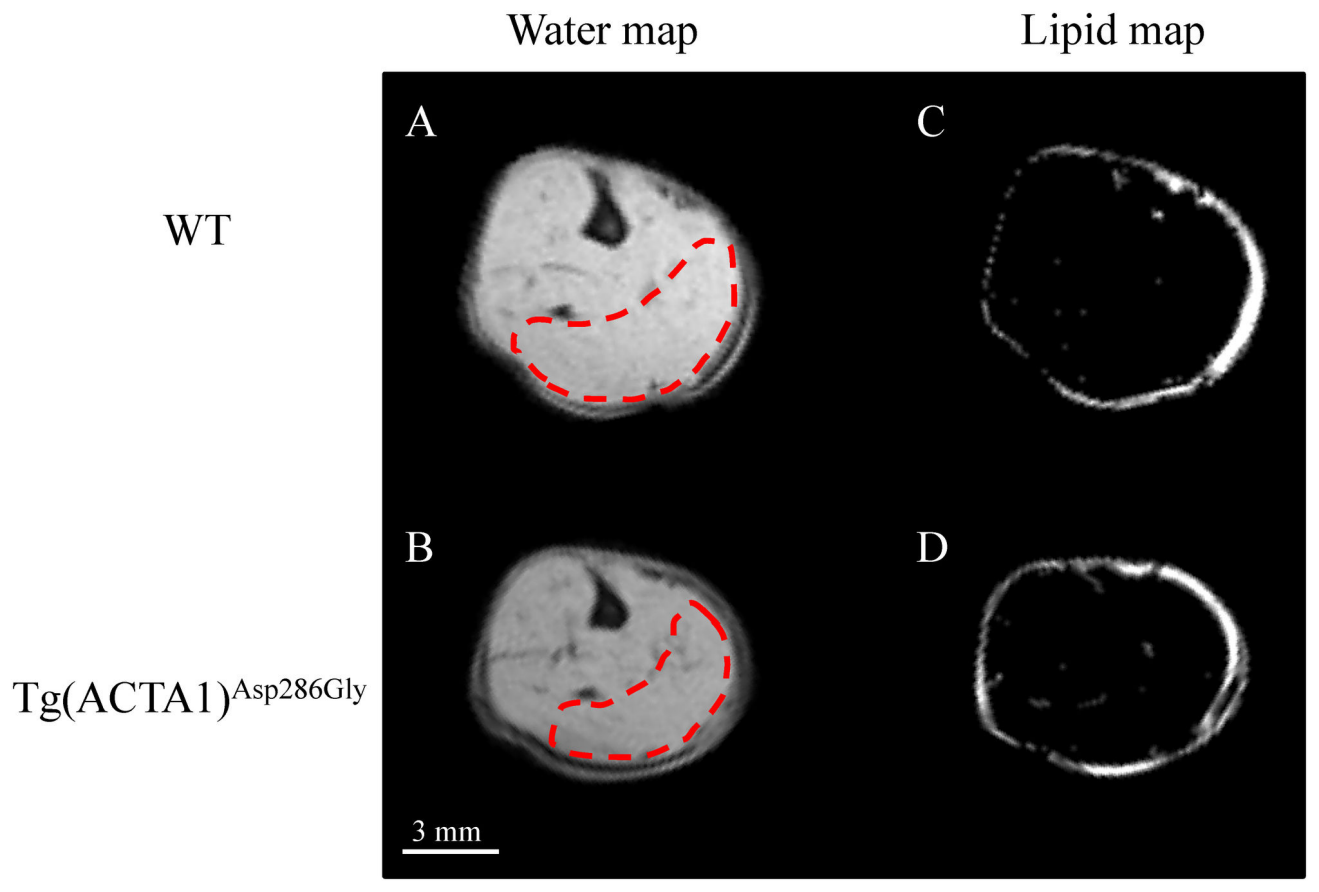
Typical representative water (A & B) and lipid (C & D) maps obtained from WT (A & C) and Tg(*ACTA1*)^Asp286Gly^ (B & D) hindlimbs. Note that intramuscular fatty infiltration was minor in both WT and Tg(*ACTA1*)^Asp286Gly^ hindlimb muscles. Bright signals in lipid maps mainly represent subcutaneous fat tissues. Regions of interest drawn in red dashes correspond to the posterior area of the hindlimb selected for T_2_ and DTI measurements.

MRI: Quantitative fat images were generated from the Dixon fat and water images (Figure 1) as follows using a home-built program developed using IDL [36]. These images were used to quantify the fat and muscle content in the whole hindlimb for the four slices with the largest sections. Fatty infiltration was calculated as follows: percentage fat = SI_fat_/(SI_fat_ + SI_water_) where SI_fat_ and SI_water_ refer to signal intensity in each pixel of the fat and water images, respectively. Maximal cross-sectional area (CSA, in mm^2^) was quantified from the largest slice from water images and the hindlimb muscles volume (in mm^3^) was calculated as the sum of the four CSAs of the five consecutive largest slices from water images obtained with the 3-point Dixon technique. T_2_ maps were generated by fitting on a pixel-by-pixel basis the logarithm of the data to the following linear equation: Log(S(TE) = Log(S_0_)-TE/T_2_ where S(TE) is the signal at time = TE and S_0_ is the equilibrium magnetization. The DTI reconstruction was performed with the manufacturer software (Bruker; Paravision 5) and the corresponding data were processed using an in-house program developed with IDL (Interactive Data Language, Research Systems Inc., Boulder, USA). DTI metrics including diffusivities (λ_1_, λ_2_ and λ_3_), apparent diffusion coefficient (ADC) and fractional anisotropy (FA) were calculated. For both T_2_ maps and DTI metrics, a region of interest was selected in the posterior area of the hindlimb (Figure 1). These measurements were done and averaged for the three slices with the largest sections.

### Statistical analyses

Statistical analyses were performed with the Statistica software version 9 (StatSoft, Tulsa, OK, USA). Normality was checked using a Kolmogorov-Smirnov test. Two-factor (group x time) analysis of variance (ANOVAs) with repeated measures on time were used to compare isometric force production, metabolites concentrations and pHi. Two-factor (group x stimulation frequency) ANOVAs with repeated measures on stimulation frequency were used to compare force production. When a main effect or a significant interaction was found, Newman–Keuls post-hoc analysis was used. Unpaired t-tests were used for other comparisons. Linear regression analysis (Pearson’s correlation) was used to compare the degree of association between variables. Data are presented as mean ± standard error of the mean (SEM). Significance was accepted when P < 0.05.

## Results

### Anatomical measurements

As illustrated in Figure 1, hindlimb muscles volume was significantly (P < 0.01) reduced by ~14% in Tg(*ACTA1*)^Asp286Gly^ mice (92 ± 3 mm^3^) as compared to WT mice (107 ± 3 mm^3^). Interestingly, a similar ~14% reduction was observed for the maximal CSA between Tg(*ACTA1*)^Asp286Gly^ mice (32.0 ± 0.9 mm^2^) and WT mice (37.2 ± 1.1 mm^2^), indicating that force normalization using either muscle volume or maximal CSA would lead to similar differences between the two groups.

The fatty infiltration was negligible for the two groups even though the magnitude was slightly higher (P < 0.001) in Tg(*ACTA1*)^Asp286Gly^ mice (0.19 ± 0.01%) versus WT mice (0.09 ± 0.01%) (Figure 1).

### Mechanical performance

**Figure 2:**
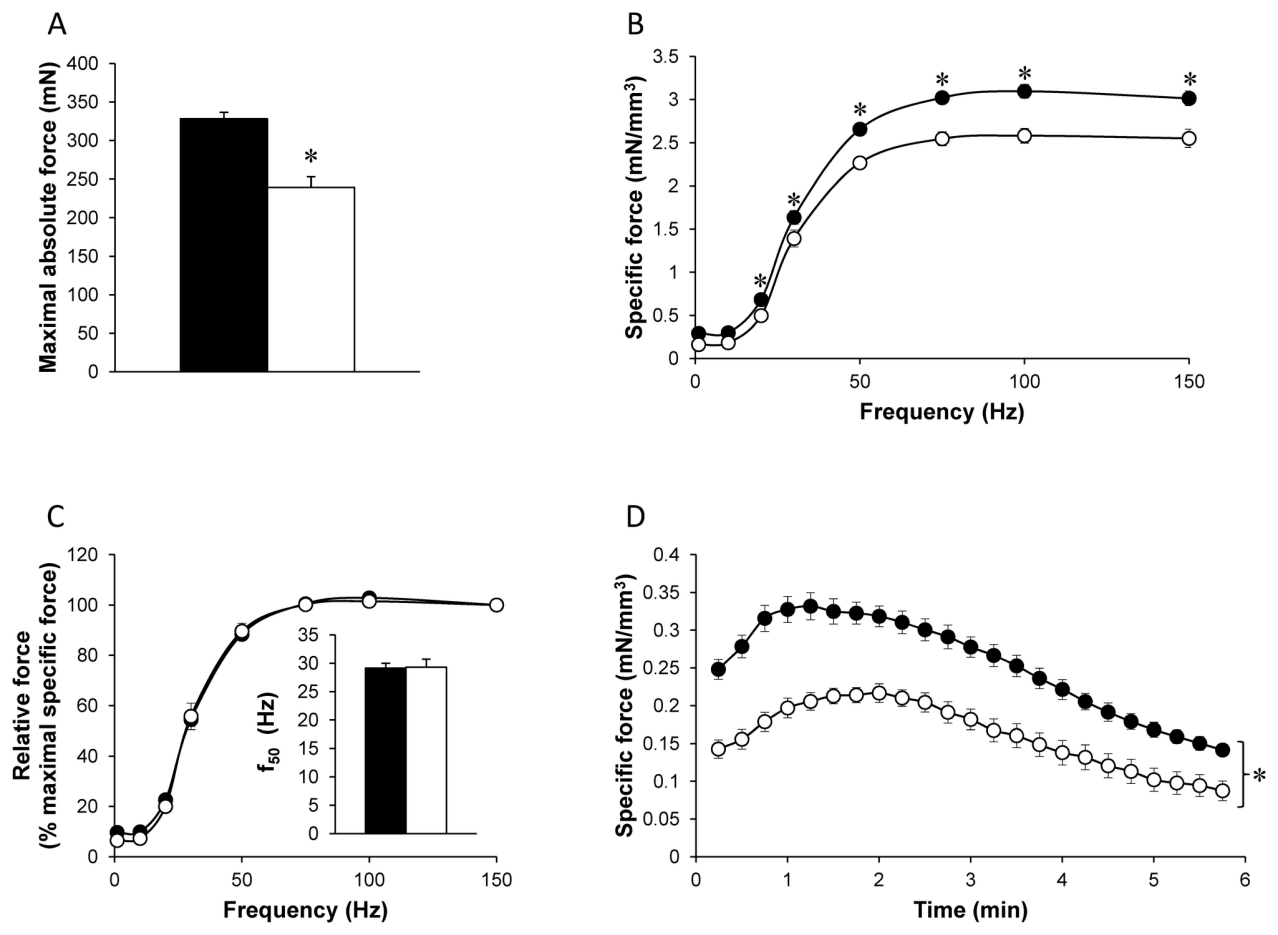
Absolute maximal force production (A), specific (B) and relative (C) force production during the force-frequency protocol and specific force production during the stimulation protocol (D) in WT group (•) and Tg(*ACTA1*)^Asp286Gly^ group (○). Force was normalized to hindlimb muscles volume (B, D) and to maximal force obtained at 150 Hz (C). f_50_ (inset Figure C) represents the frequency resulting in half the maximal force production. Absolute (A) and specific force production (B & D) were lower in Tg(*ACTA1*)^Asp286Gly^ group as compared to the WT group. On the contrary, no difference was observed for f_50_ between the two groups. Values are presented as the mean ± SEM. Significantly different between groups * *P* < 0.05.

As can be seen in Figure 2A, we measured a significantly reduced absolute maximal tetanic force in Tg(*ACTA1*)^Asp286Gly^ mice (-30%; P < 0.001) relative to WT mice. The specific tetanic force was also significantly lower (P < 0.001) in Tg(*ACTA1*)^Asp286Gly^ mice for stimulation frequencies ranging from 20 to 150 Hz so that maximal specific tetanic force was reduced by 15% in Tg(*ACTA1*)^Asp286Gly^ mice (Figure 2B). Interestingly, the maximum rate of force development was slower (P < 0.001) in the Tg(*ACTA1*)^Asp286Gly^ mice (2.42 ± 0.15 mN/ms) as compared to WT mice (3.15 ± 0.09 mN/ms), while the half relaxation time was shorter (P < 0.01) in Tg(*ACTA1*)^Asp286Gly^ mice (148 ± 21 ms) in comparison with WT mice (262 ± 26 ms). In order to take into account the differences in specific force production between the two groups, a relative force-frequency curve was constructed using force values expressed as a percentage of the maximally generated force at 150 Hz. As illustrated in Figure 2C, these curves were similar and the corresponding f_50_ values were identical (Inset Figure 2C). Force production during the standardized 6-min stimulation protocol was also significantly lower (P < 0.001) in the Tg(*ACTA1*)^Asp286Gly^ group (Figure 2D).

### Metabolic changes

**Figure 3:**
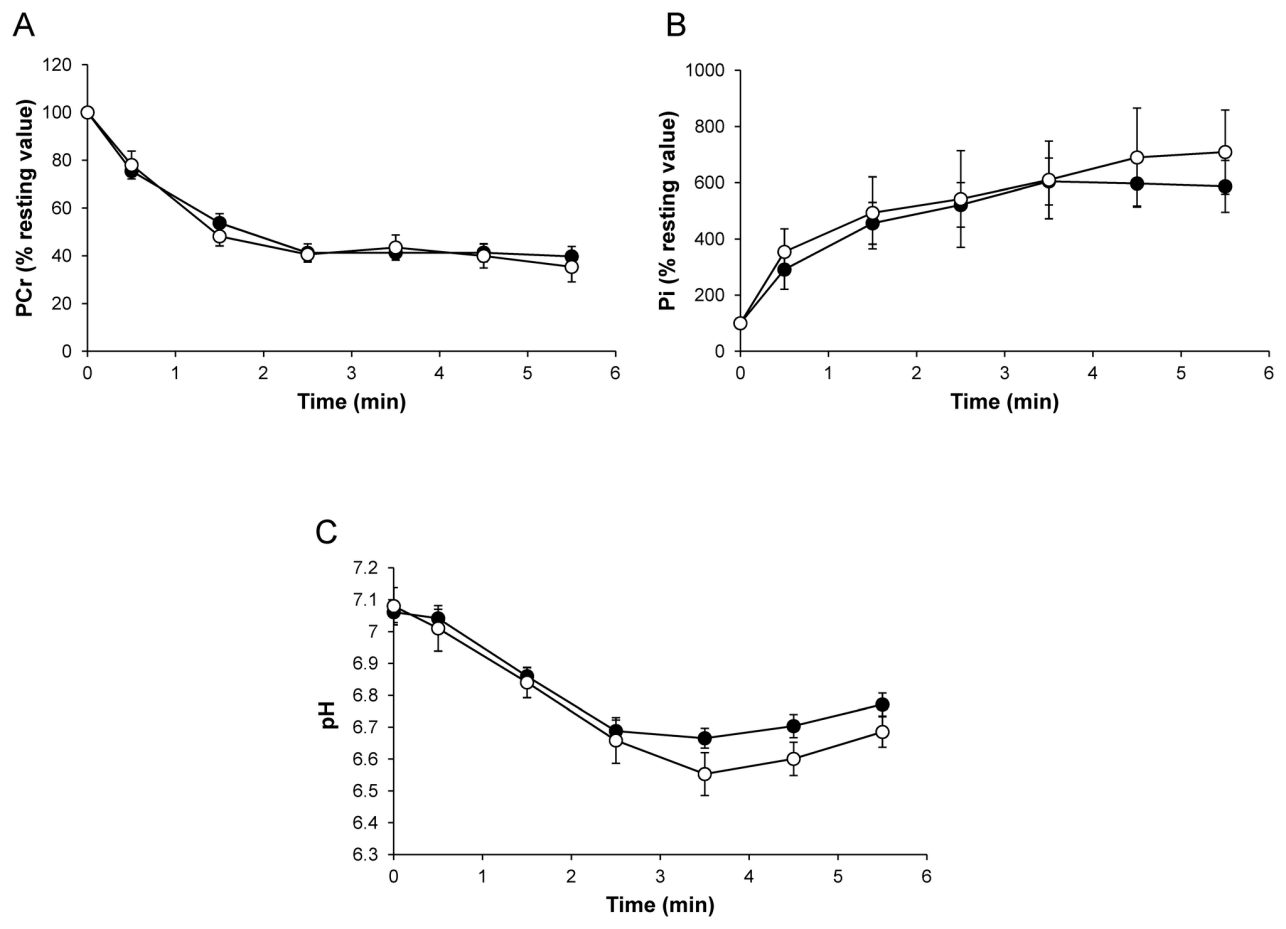
Changes in gastrocnemius PCr (% resting value; A), Pi (% resting value; B), and pHi (C) during the fatiguing protocol were similar between WT (•) and Tg(*ACTA1*)^Asp286Gly^ groups (○). Values are presented as mean ± SEM.

[PCr]/[ATP] resting ratios were not significantly different (P > 0.05) between Tg(*ACTA1*)^Asp286Gly^ (3.0 ± 0.2) and WT groups (3.5 ± 0.4). For both groups, [PCr] fell rapidly throughout the fatigue protocol and reached a steady state at the end of the stimulation bout. No significant difference (P > 0.05) was observed between the two groups throughout the stimulation period (Figure 3A). As expected, the [Pi] time-course evolved as a mirror of the [PCr] time-dependent changes. For both groups, [Pi] increased during the fatigue protocol and reached a plateau after 3 min of exercise (Figure 3B). At rest, pHi was not significantly different (P > 0.05) in WT (7.06 ± 0.03) and Tg(*ACTA1*)^Asp286Gly^ mice (7.08 ± 0.06). pHi decreased throughout the stimulation session so that the acidosis extent was similar for the two groups at the end of the fatigue protocol (Figure 3C). Taken together, the fatigue protocol-induced metabolic changes were comparable between the two groups. However, given that the force production was significantly lower in Tg(*ACTA1*)^Asp286Gly^ mice, one could suggest that their energy cost of contraction was increased. No significant difference (P > 0.05) was observed for τPCr between the two groups of mice (148 ± 9 sec *vs.* 159 ± 11 sec for WT and Tg(*ACTA1*)^Asp286Gly^ mice, respectively).

### T_2_ and DTI metrics

**Figure 4:**
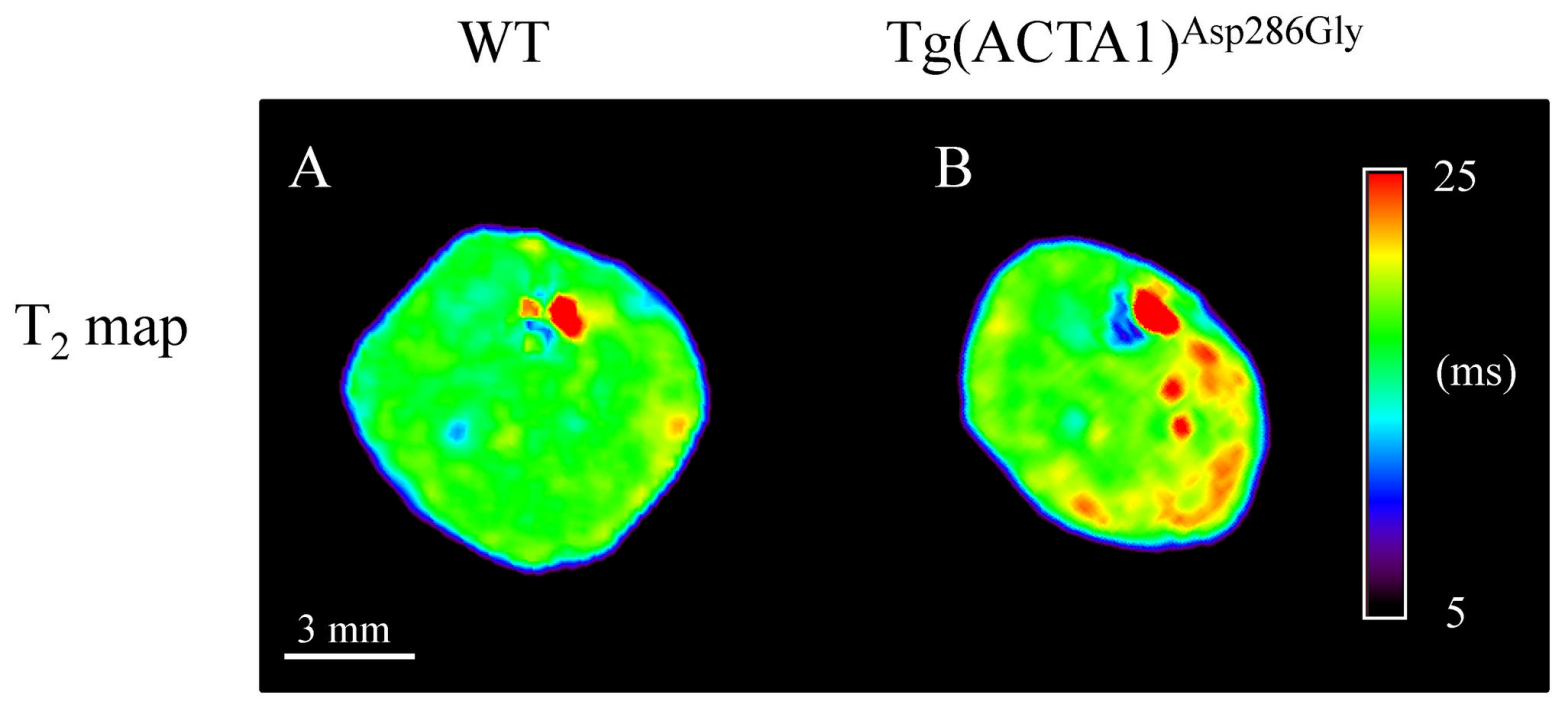
Typical representative T_2_ maps obtained from WT (A) and Tg(ACTA1)^Asp286Gly^ (B) hindlimbs. Note that T_2_ was significantly higher in the posterior area for Tg(*ACTA1*)^Asp286Gly^ muscles as compared to WT muscles.

**Figure 5:**
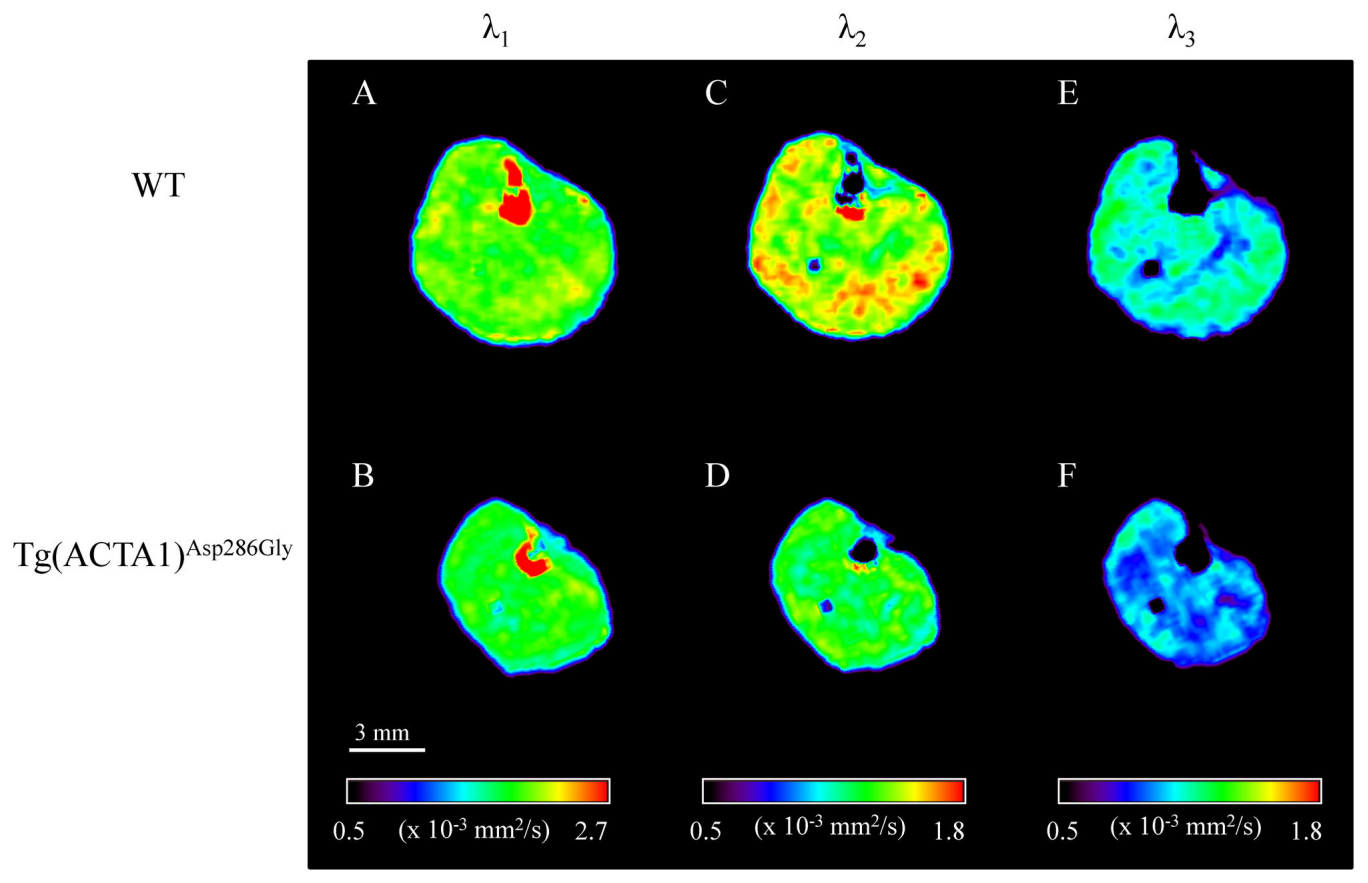
Typical representative λ_1_ (A & B), λ_2_ (C & D) and λ_3_ (E & F) maps obtained from WT (A, C & E) and Tg(*ACTA1*)^Asp286Gly^ (B, D & F) mice hindlimbs. Note that λ_2_ and λ_3_ were significantly lower in the posterior area for Tg(*ACTA1*)^Asp286Gly^ while λ_1_ was unaltered.

**Figure 6:**
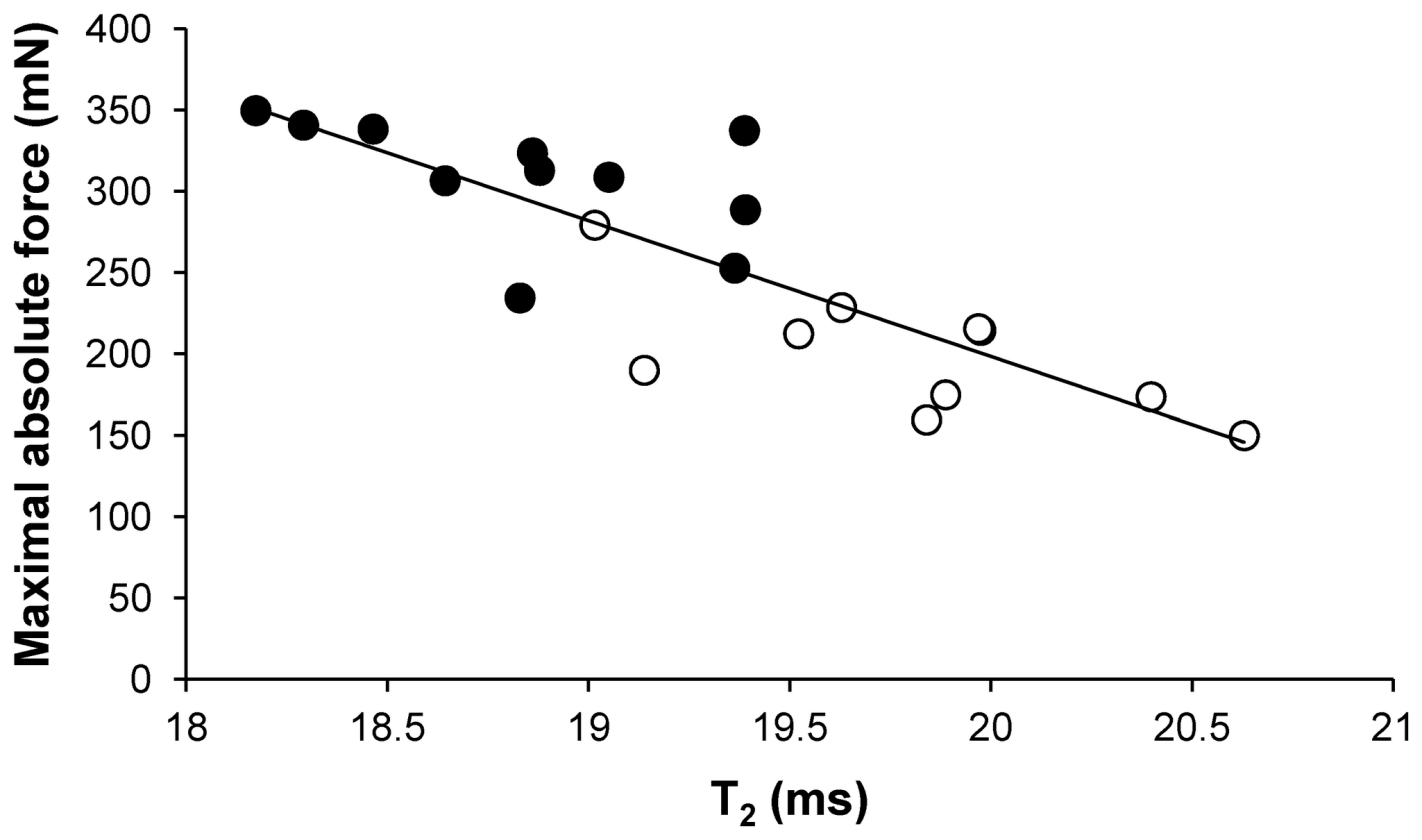
Linear relation between T_2_ values and maximal absolute force in the WT(•) and Tg(*ACTA1*)^Asp286Gly^ group (○). Note that the higher the T_2_, the lower the force production. r² = 0.70; P < 0.05.

T_2_ values were significantly higher in Tg(*ACTA1*)^Asp286Gly^ mice as compared to WT mice (+ 5%, P < 0.001) (Table 1 & Figure 4). Furthermore, λ_2_, λ_3_ and ADC were significantly lower (P < 0.05) in the Tg(*ACTA1*)^Asp286Gly^ cohort when compared to the cohort of WT mice (-8%, -7% and -6%, respectively; Figure 5). On the contrary, both λ_1_ and FA were not significantly different (P > 0.05) between the two groups (Table 1 & Figure 5). A significant negative correlation (P < 0.05) was found between T_2_ values and maximal absolute force (*r* ^2^ = 0.70; Figure 6). Both λ_2_ (*r* ^2^ = 0.39) and λ_3_ (*r* ^2^ = 0.39) were positively correlated (P < 0.05) to muscle volume of the posterior area of the hindlimb.

**Table 1:** Mean T_2_ values, apparent diffusion coefficient (ADC), fractional anisotropy (FA) and the three eigenvalues obtained from WT and Tg(*ACTA1*)^Asp286Gly^ mice.

	**WT**	**Tg(*ACTA1*)^Asp286Gly^**
**T_2_ (ms)**	18.8 ± 0.1	19.8 ± 0.2*
**ADC (x 10^-3^ mm^2^/s)**	1.49 ± 0.04	1.41 ± 0.02*
**FA**	0.29 ± 0.01	0.31 ± 0.01
**λ_1_ (x 10^-3^ mm^2^/s)**	1.95 ± 0.04	1.88 ± 0.06
**λ_2_ (x 10^-3^ mm^2^/s)**	1.45 ± 0.04	1.34 ± 0.02*
**λ_3_ (x 10^-3^ mm^2^/s)**	1. 07 ± 0.03	1.00 ± 0.02*

Values are presented as the mean ± SEM. Significantly different from WT group * *P* < 0.05.

## Discussion

Considering the rarity of NM, the well-known limitations linked to the analysis of biopsy samples and the possible differences between *in vitro* and *in vivo* measurements of muscle function, we aimed at investigating *in vivo* the functional, anatomical and metabolic consequences of the *ACTA1* Asp286Gly mutation in a recently generated transgenic NM mouse model by utilising a strictly noninvasive approach. We observed an impaired maximal force production in Tg(*ACTA1*)^Asp286Gly^ mice which may be partially related to muscle atrophy. T_2_ values and DTI metrics were also modified in Tg(*ACTA1*)^Asp286Gly^ mice. Interestingly, muscle weakness was linearly related to T_2_ values and was associated to an increased energy cost of contraction.

For the first time, *in vivo* hindlimb muscle function in Tg(*ACTA1*)^Asp286Gly^ mice was characterized. We showed that absolute maximal tetanic force was largely reduced, i.e. ~ 30%, in Tg(*ACTA1*)^Asp286Gly^ mice as compared to controls. We also observed by Dixon MRI that hindlimb muscles volume of Tg(*ACTA1*)^Asp286Gly^ was reduced by ~ 15%, thereby illustrating that the Asp286Gly mutation leads to muscle atrophy, a typical feature of NM patients [37,38]. It should be pointed out that the specific force (taking into account the reduced muscle volume) was still ~ 15% lower in Tg(*ACTA1*)^Asp286Gly^ mice, indicating that muscle weakness can be related to physiological processes other than muscle atrophy alone. Accordingly, a recent study showed that the lower force production of Tg(*ACTA1*)^Asp286Gly^ mice was related to a smaller number of myosin cross-bridges strongly bound to actin monomers [13]. On that basis, our findings indicate that the poisoning effect of the Asp286Gly mutation also affects *in vivo* skeletal muscle function, leading to a mild pathological contractile phenotype. However, it should be pointed out that the 15% reduction in maximal specific force we measured *in vivo* was smaller than the corresponding changes measured *in vitro*, i.e. ~ a 40-45% reduction in both isolated muscles and single muscle fibers from Tg(*ACTA1*)^Asp286Gly^ mice [10,13]. Although *in vivo* and *in vitro* experiments were performed on two different muscles (gastrocnemius and EDL muscles respectively), considering that their fiber type composition is similar, the different contractile impairment might not be related to the muscle typology. Interestingly, a similar finding has been reported in *Acta1*(H40Y) mice with a larger muscle weakness identified *in vitro* (~ 40%) [8] as compared to *in vivo* (~ 25%) [32]. Taken together, our findings indicate that NM-induced muscle weakness might be milder *in vivo* than *in vitro* and further studies are warranted to unravel the underlying mechanisms.

Given that the force-frequency curves we obtained in Tg (*ACTA1*)^Asp286Gly^ and WT mice were similar, our result does not support the reduced calcium sensitivity previously reported on the basis of the rightward shift of both the force-frequency and the force-Ca^2+^ curves obtained in isolated EDL muscles and single fibers from Tg(*ACTA1*)^Asp286Gly^ mice [10]. Surprisingly, a leftward shift of the force-frequency curve, indicating either a higher calcium sensitivity or a slower cross-bridges kinetic, was reported in EDL muscle when EGFP-tag was included in Tg(*ACTA1*)^Asp286Gly^ mice [11] so that the effects of the Asp286Gly mutation on calcium sensitivity remain unclear. Our findings are in line with those recently reported in *Acta1*(H40Y) mice for which the force-frequency relationship was not modified *in vivo* as compared to controls [32]. Although it has been recently suggested that Ca^2+^ sensitizing agents might counterbalance muscle weakness in NM patients carrying mutations in the *NEB* gene [39,40], our data indicated that these pharmaceutical agents would be ineffective for counteracting the deleterious effects of the Asp286Gly mutation on *in vivo* muscle function.

Using the Dixon technique, we accurately quantified the potential fatty infiltration in hindlimb muscles of the Tg(*ACTA1*)^Asp286Gly^ mice. On the contrary to what has been reported in NM patients carrying *ACTA1* mutations i.e. a progressive replacement of skeletal muscle by fatty tissue [37,41], the intramuscular fat content was negligible in Tg(*ACTA1*)^Asp286Gly^ muscles (< 1%). These findings are in line with those recently reported in *Acta1*(H40Y) mice which also displayed minor intramuscular fat content as compared to controls [32]. Overall, the present Dixon MRI data indicated that the Tg(*ACTA1*)^Asp286Gly^ mouse model did not reproduce the large fatty infiltration sometimes observed in NM patients. Interestingly, a similar finding was reported in muscles of *mdx* mice [42,43] as compared to boys with Duchenne muscular dystrophy [44]. It remains to be determined whether the lack of fatty infiltration in NM mouse muscles is due to a lower degree of degeneration and/or to an improved regenerative process as compared to NM patients.

T_2_ mapping revealed higher mean T_2_ values in Tg(*ACTA1*)^Asp286Gly^ as compared to WT mice. It has been well acknowledged that T_2_ changes occur in a variety of pathophysiological events (e.g. edema, necrosis, inflammation and fatty infiltration) [24,25]. Considering the reduced hindlimb muscle volume and the absence of fatty infiltration in Tg(*ACTA1*)^Asp286Gly^ mice, the increased T_2_ is unlikely to be related to these two features. Therefore the elevated muscle T_2_ in Tg(*ACTA1*)^Asp286Gly^ mice could account for muscle degeneration/regeneration of myofibers as illustrated by the presence of internally-nucleated muscle fibers in Tg(*ACTA1*)^Asp286Gly^ mice with the EGFP-tag [10]. Interestingly, we found a strong and negative correlation between T_2_ values and maximal force, indicating that the higher the T_2_, the lower the force production. Although a similar correlation between T_2_ and clinical assessments has been recently observed in dystrophic patients, the corresponding T_2_ values were also related to fatty infiltration [23], thereby precluding differentiation between T_2_ increases due to damaged muscle fibres and elevated T_2_ due to fat infiltration. Quite the reverse, fat infiltration was negligible in Tg(*ACTA1*)^Asp286Gly^ mice and more importantly we used a fat suppression module for T_2_ MRI acquisitions. As a consequence, the presence of damaged/regenerated muscle fibers, as illustrated by higher T_2_ values, might contribute to the impaired mechanical performance of Tg(*ACTA1*)^Asp286Gly^ mice. On that basis, T_2_ mapping could be a relevant tool for monitoring muscle weakness and disease severity in NM patients.

Diffusion-weighted MRI provides information about self-diffusion of water molecules, which is restricted by physical barriers such as membranes, cytoskeleton, mitochondria and sarcoplasmic reticulum, thereby leading to anisotropic diffusion. Surprisingly, the large histological spectrum of the Tg(ACTA1)^Asp286Gly^ mice did not modify water diffusivity along the long axis of muscle fibers as illustrated by the unchanged values of λ_1_. In contrast, we showed that both λ_2_ and λ_3_ values were lower in the Tg(ACTA1)^Asp286Gly^ group as compared to controls, leading to a reduced ADC values in transgenic mice. We found that muscle volume of the posterior area of the hindlimb was positively correlated to both λ_2_ and λ_3_ so that the lower the muscle volume, the lower the eigenvalues. Accordingly, our DTI experiments support previous results suggesting that the second and third eigenvalues might be considered as markers of myofiber atrophy [45,46]. As a consequence, these two eigenvalues might be a relevant tool for strictly noninvasively monitoring skeletal muscle atrophy in NM patients. Alternatively, the histological features such as nemaline rods, tubular aggregates or ringbinden fibers might also hinder water diffusion in the radial axis and contribute to the lower values of λ_2_ and λ_3_ in Tg(*ACTA1*)^Asp286Gly^ mice. Consequently, multimodal MRI investigations might provide relevant biomarkers for monitoring the severity and/or the progression of NM due to skeletal muscle actin mutations.

^31^P-MRS investigations showed that PCr consumption, Pi production and acidosis were not different between the two groups throughout the stimulation protocol. However, the force production was lower in the Tg(*ACTA1*)^Asp286Gly^ mice as compared to the WT mice, especially during the two first minutes of the exercise session, i.e. where the ATP production was mainly related to anaerobic processes (i.e., including PCr depletion and glycolysis). On that basis, one could suggest that the anaerobic cost of contraction was higher in the Tg(*ACTA1*)^Asp286Gly^ mice as compared to controls, a finding similar to what has been recently reported in the *Acta1*(H40Y) mice model [32]. Although ATP production through oxidative pathways cannot be directly measured during exercise, it has been proposed that the analysis of PCr recovery kinetics is a relevant mirror of the end-exercise rate of oxidative ATP synthesis. We observed that τPCr values were not different between the two groups thereby suggesting that mitochondrial capacity was preserved in the Tg(*ACTA1*)^Asp286Gly^ mice and that the oxidative ATP production was not impaired by the mutation. Overall, the increased energy cost of contraction in transgenic mice may be related to a higher anaerobic cost. It should be emphasized that during muscular activity, ATP is used by both contractile and non-contractile processes [47,48]. Interestingly, Ochala et al. recently reported an unchanged rate constant of force redevelopment and an increased maximal unloaded shortening velocity in Tg(*ACTA1*)^Asp286Gly^ mice, thereby indicating that the Asp286Gly mutation would lead to a reduced rate of cross-bridge attachment and an increased rate of cross-bridge detachment. Thus the increased energy cost in Tg(*ACTA1*)^Asp286Gly^ mice might be related, at least in part, to an alteration occurring at the cross-bridge level, a finding consistent with our observations of a slower rate of force development and a shorter half-relaxation time observed in Tg(*ACTA1*)^Asp286Gly^ mice. Alternatively, the higher energy cost in transgenic mice might also be associated with a higher rate of ATP utilization by non-contractile processes. Indeed, analysis of Tg(*ACTA1*)^Asp286Gly^ mouse muscles revealed the presence of tubular aggregates, structures that are mainly composed of key proteins involved in the uptake and storage of calcium [49,50]. Interestingly, a higher cytoplasmic Ca^2+^ level has been recently reported in myoblasts from a patient with tubular-aggregate myopathy [51]. This finding has been related to an excessive extracellular Ca^2+^ influx into the cytoplasm due to an impairment of Ca^2+^ sensing in the sarcoplasmic reticulum. One could thereby speculate that ATP would be inevitably needed to remove this calcium excess out of the cytosol of the Tg(*ACTA1*)^Asp286Gly^ muscle fibers in order to avoid a large dysregulation of Ca^2+^ homeostasis and ultimately to limit/prevent muscle fiber necrosis [52]. Further studies would be warranted in order to improve our understanding on the influence of tubular aggregates on muscle energetics.

In conclusion, our exclusively noninvasive methodological approach provides compelling evidence of an altered *in vivo* muscle function in Tg(*ACTA1*)^Asp286Gly^ mice. The presence of histological anomalies and muscle atrophy in Tg(*ACTA1*)^Asp286Gly^ mice might alter both T_2_ values and DTI metrics that might be considered as relevant biomarkers for monitoring the severity and/or the progression of this disease, but also for assessing the efficacy of potential therapeutic interventions at a preclinical level.
